# Antiviral Effect of pIFNLs against PEDV and VSV Infection in Different Cells

**DOI:** 10.3390/ijms23179661

**Published:** 2022-08-26

**Authors:** Jing Chen, Wang Xu, Peiheng Li, Lina Song, Yuhang Jiang, Pengfei Hao, Zihan Gao, Wancheng Zou, Ningyi Jin, Chang Li

**Affiliations:** 1 Key Laboratory for Zoonosis Research, Ministry of Education, College of Veterinary Medicine, Jilin University, Changchun 130062, China; 2 Research Unit of Key Technologies for Prevention and Control of Virus Zoonoses, Chinese Academy of Medical Sciences, Changchun Veterinary Research Institute, Chinese Academy of Agricultural Sciences, Changchun 130122, China

**Keywords:** pIFNLs, pIFNα, VSV-EGFP, PEDV, antiviral activity

## Abstract

Type III and type I interferon have similar mechanisms of action, and their different receptors lead to different distributions in tissue. On mucosal surfaces, type III interferon exhibits strong antiviral activity. Porcine epidemic diarrhea virus (PEDV) is an economically important enteropathogenic coronavirus, which can cause a high incidence rate and mortality in *piglets*. Here, we demonstrate that porcine interferon lambda 1 (pIFNL1) and porcine interferon lambda 3 (pIFNL3) can inhibit the proliferation of vesicular stomatitis virus with an enhanced green fluorescent protein (VSV-EGFP) in different cells, and also show strong antiviral activity when PEDV infects Vero cells. Both forms of pIFNLs were shown to be better than porcine interferon alpha (pIFNα), the antiviral activity of pIFNL1 is lower than that of pIFNL3. Therefore, our results provide experimental evidence for the inhibition of PEDV infection by pIFNLs, which may provide a promising treatment for the prevention and treatment of Porcine epidemic diarrhea (PED) in *piglets*.

## 1. Introduction

The first interferon (IFN) described in modern medical research was type I IFN. Interferon plays an important role in the innate immune systems of vertebrates and has gained much attention in modern medical research [1]. The biological functions include the modulation of innate and adaptive immune responses, anti-proliferative effects, and most importantly, antiviral properties [2,3].

The type III IFNs or IFNLs were described along with type I IFNs [4,5]. IFNLR1 and IL-10R2 are heterodimeric receptors that are used by type III IFNs to signal, whereas IL-28R1 is used by type I IFNs [6,7,8]. The expression of IFNLR1 is not ubiquitous but appears to be limited to epithelial cells and certain immune cells [9,10,11,12,13]. Throughout history, the limited tropism of the type III IFN receptor has led to the hypothesis that type III IFNs exhibit unique properties on mucosal surfaces [13,14,15,16,17,18,19,20].

While type I and type III IFNs differ from sequence and structural perspectives, as well as using two different receptor complexes, cytokines induce a remarkably similar panel of interferon-stimulated genes (ISGs) in response to interferons. In the first instance, the binding of type I and III IFNs to IFN receptors induces conformational changes in the intracellular part. JAK1, JAK2, and TYK2 (Janus kinase) will be activated by IFN binding. In response to this activation, signal transducer and activator of transcription (STAT) proteins are recruited, which are then phosphorylated by JAKs; IFN-stimulated gene factor 3 (ISGF3) is formed and combined with IRF9 to form a complex. After translocation to the nucleus, the complex regulates ISG expression [21,22,23,24,25,26,27,28,29].

Although there are more studies on type III interferon, there are relatively few studies on animal interferon. In this study, porcine type III interferons (pIFNLs) were prepared using the baculovirus expression system. Using porcine type I interferons (pIFNα) as a positive control, the antiviral activities of pIFNLs were analyzed. The results showed that pIFNLs could not only activate a group of IFN-regulated genes but also significantly inhibit the replication of VSV-EGFP and PEDV in cells. pIFNL3 had the highest antiviral activity, followed by pIFNL1, and the antiviral activity of pIFNα was the lowest. In conclusion, we demonstrated that pIFNLs and pIFNα displayed robust antiviral activity against VSV-EGFP and PEDV infection. Moreover, pIFNLs preferably provide critical antiviral defenses compared to pIFNα.

## 2. Results

### 2.1. Preliminary Analysis of the Antiviral Activities of pIFNLs

**Figure 1:**
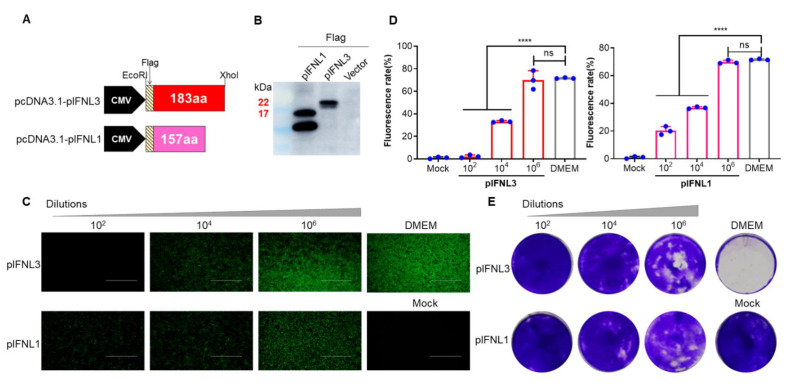
Preliminary analysis of antiviral activities of pIFNLs. (**A**) Schematics of the two recombinant plasmids pcDNA3.1-pIFNLs. (**B**) Expression of pIFNLs identified by WB. (**C**) The identified antiviral activities with different dilution times of IFNLs were analyzed by fluorescence observation using 12-well plates 12 h after VSV-EGFP (0.01 MOI) infection. The scale bar corresponds to 750 μm. (**D**) The identified antiviral activities with different dilution times of IFNLs were analyzed by flow cytometry using 12-well plates 12 h after VSV-EGFP (0.01 MOI) infection. ****, *p* < 0.0001; ns, no significant difference. (**E**) The identified antiviral activities with different dilution times of IFNLs were analyzed by crystal violet stain 36 h after VSV-EGFP (0.01 MOI) infection.

To assess the antiviral activities of pIFNLs, the plasmid containing pIFNL1 and pIFNL3 were constructed based on pcDNA3.1 (Figure 1A). Transfecting human embryonic kidney cells (HEK293) with these constructs, the empty Vector and Mock served as negative controls. The WB result showed that pIFNL1 and pIFNL3 were expressed correctly, and a slightly smaller target band appeared in pIFNL1, which may have been caused by incomplete glycosylation (Figure 1B). Then the cell supernatant was collected after 48 h, and madin-Darby canine kidney (MDCK) cells seeded in 12-well plates (Corning, Corning, NY, USA) were treated for 12 h with serial dilutions of pIFNL preparations diluted 100-fold in DMEM. Cells were infected with VSV-EGFP at a multiplicity of infection (MOI) of 0.01. Fluorescence observation and flow cytometry were performed after 12 h. The results showed that pIFNL1 and pIFNL3 blocked VSV-EGFP infection significantly (Figure 1C,D). Furthermore, the proliferation of VSV-EGFP in MDCK cells was evaluated using the crystal violet staining assay (Figure 1E). The cell viability was significantly increased in the pIFNL-treated cells compared with the control groups. Moreover, with the increase of the dilution ratio, the virus infection ability became stronger, and the cell viability decreased obviously. The results show that pIFNL1 and pIFNL3 have antiviral activities and the inhibitory effect on the virus is dose-dependent.

### 2.2. Generation of the Recombinant Baculoviruses rBV-pIFNL1, rBV-pIFNL3

**Figure 2:**
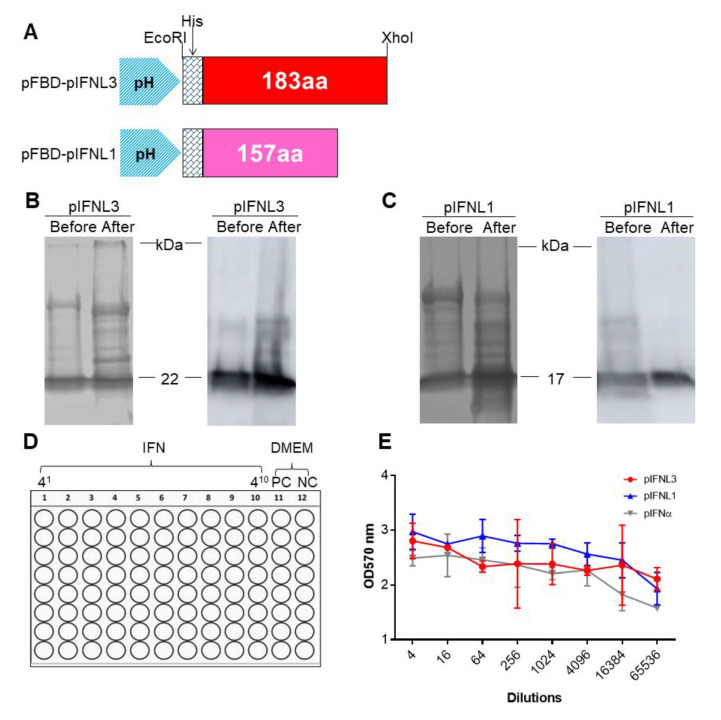
Expressions of pIFNLs by the baculovirus expression system and antiviral activity analysis, and pIFNα served as the control. (**A**) Schematics of the two recombinant shuttle plasmids pFBD-pIFNLs. (**B**,**C**) Expression of pIFNLs identified by SDS-PAGE and WB. “Before” means unpurified protein of pIFNLs. “After” means purified protein of pIFNLs. (**D**) The protocols for detecting the biological activity of pIFNLs. “PC” means positive control of the cells treated only with VSV-EGFP but not IFNs. (**E**) The OD570 nm value was used to evaluate the potency of interferon.

To further evaluate the biological properties of pIFNLs, the baculovirus expression system (BES) was used to prepare the target protein. The pIFNL gene was optimized, synthesized, and subcloned into the PH promotor of the shuttle pFastBac™ 1 vector according to the protocols described previously [30]; pIFNα (gifted from XIANPUAIRUI SCIENCE, Gaomi, Shandong, China) served as a positive control (Figure 2A). The proteins were purified by ultracentrifugation with sucrose cushions. The SDS-PAGE assay and western blot showed that the target proteins were prepared successfully (Figure 2B,C). Then, the biological activities of pIFNLs and pIFNα were analyzed by a VSV-EGFP infection experiment according to the protocols (Figure 2D). The results of crystal violet staining showed that pIFNL1, pIFNL3, and pIFNα had biological activities (pIFNL1, 1.5 × 10^4^ IU/0.1 mL; pIFNL3, 1.3 × 10^4^ IU/0.1 mL; pIFNα, 1.28 × 10^3^ IU/0.1 mL), and the antiviral effect was dose-dependent (Figure 2E).

### 2.3. pIFNLs Inhibits VSV-EGFP Proliferation

**Figure 3:**
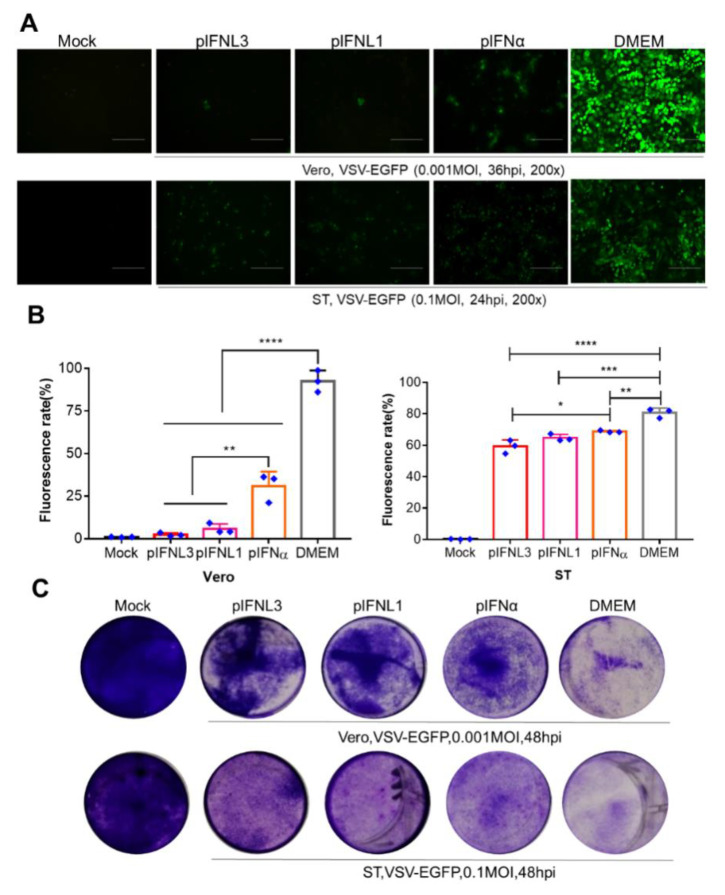
Antiviral activities of pIFNLs against VSV-EGFP in ST and Vero cells; pIFNα served as the control. (**A**) The identified antiviral activities of IFNLs were analyzed by crystal violet after VSV-EGFP infection. Mock means the normal cells without any treatment. The scale bar corresponds to 150 μm. (**B**,**C**) Analysis of the antiviral activity of pIFNL1 by fluorescence observation and flow cytometry using 12-well plates after VSV-EGFP infection. *, *p* < 0.05; **, *p* < 0.01; ***, *p* < 0.001; ****, *p* < 0.0001.

To further determine whether IFNs had antiviral effects on other cells, both swine testis cells (ST) and african green monkey kidney cells (Vero) were treated with IFNs and infected with VSV-EGFP after 12 h to analyze the antiviral effects. The results showed that pIFNL1, pIFNL3, and pIFNα blocked the VSV-EGFP infection significantly (Figure 3A,B). Furthermore, the proliferation of VSV-EGFP in cells was evaluated using the crystal violet staining assay, and the cell viability in the interferon-treated group was higher than that in the control group (Figure 3C). Moreover, pIFNL3 had the highest antiviral activity, followed by pIFNL1; pIFNα had the worst effect.

### 2.4. pIFNLs Inhibits PEDV Proliferation in Vero Cells

**Figure 4:**
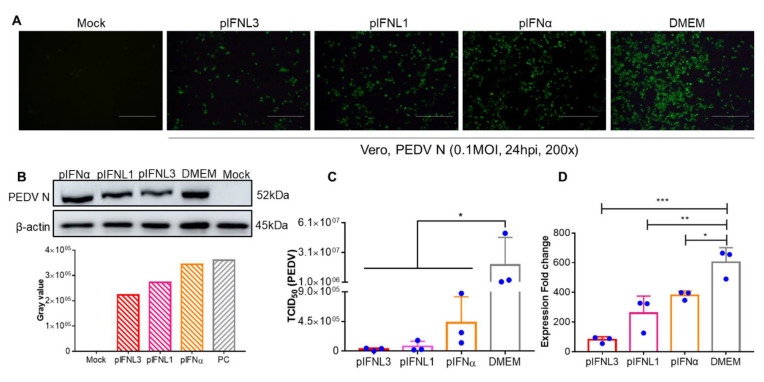
Antiviral activities of pIFNLs against PEDV in Vero; pIFNα served as the control. (**A**) Analysis of the antiviral activities of pIFNLs by IFA. The scale bar corresponds to 150 μm. Using the PEDV N protein, specific antibodies as the primary antibodies, and FITC-labeled goat anti-rabbit IgG (H + L) as the secondary antibody, the virus protein was detected. (**B**) Analysis of antiviral activities of pIFNLs by WB. Using the PEDV N protein, specific antibodies as the primary antibodies, and HRP-labeled goat anti-rabbit IgG (H + L) as the secondary antibody, the virus protein was detected. (**C**) Analysis of the antiviral activities of pIFNLs by TCID_50_. (**D**) Analysis of antiviral activities of pIFNLs by qPCR. *, *p* < 0.05; **, *p* < 0.01; ***, *p* < 0.001.

To determine whether pIFNLs could inhibit PEDV proliferation, Vero cells were treated with pIFNLs (100 IU/mL) for 12 h followed by infection with PEDV at a multiplicity of infection (MOI) of 0.01; pIFNα was the positive control. IFA and WB were performed after 24 hpi to analyze the expression of the PEDV N protein (Figure 4A,B). The results showed that pIFNL1, pIFNL3, and pIFNα inhibited PEDV infection significantly. The proliferation of PEDV was evaluated using viral titers and qRT-PCR (Figure 4C,D); the results showed consistency with IFA and WB results. The above results show that both type I and type III interferons can inhibit the proliferation of PEDV on Vero cells, but there are differences in antiviral activities. Of the two type III interferons, pIFNL3 has higher antiviral activity.

### 2.5. pIFNLs Induced ISGs Production

**Figure 5:**
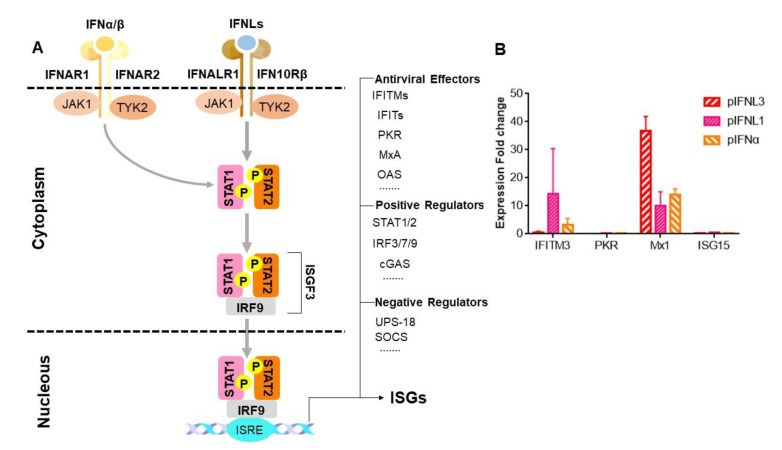
pIFNLs induced ISGS production. (**A**) Pattern diagram of ISG induction, induced by IFN. IFN interacts with the IFN receptor complex and initiates the formation of the ISGF3 transcription factor through the JAK-STAT pathway. The ISG induction, which determines the host’s antiviral state, depends on the ISGF3 nuclear translocation and interaction with the ISRE promoter. (**B**) Transcriptional profiling of the IFITM3, double-stranded RNA-dependent protein kinase (PKR), Mx1. and interferon-stimulated gene 15 (ISG15) genes after the pIFNL-treated ST cells. The shown fold induction is the average performed in triplicate.

As we know, the receptor complex initiates the janus kinase (JAK)-signal transducer and activator of transcription (STAT) signaling pathways (JAK-STAT signal pathway) by binding to the common receptors and finally activates ISGF3. ISGF3 translocation leads to the transcriptional activation and massive expression of ISGs through the ISRE promoter to establish an antiviral state; IFNL exerts antiviral effects by inducing ISGs (Figure 5A). To demonstrate whether pIFNLs have similar mechanisms, we incubated pIFNLs with ST cells, which could stimulate ISG expression (Figure 5B). The results showed that pIFNLs upregulated the expressions of several antiviral proteins, including myxovirus resistant 1 (Mx1) and interferon-induced transmembrane (IFITM3).

## 3. Discussion

In response to viral infection, interferons are among the most important molecules of innate immunity [31,32,33]. PEDV, an enteropathogenic alpha coronavirus, is responsible for swine diseases with high economic implications [34]. Acute malabsorption syndrome with watery diarrhea is caused by PEDV infection of the small intestinal epithelial cells in vivo, with symptoms of vomiting and anorexia in any age of a *pig*. Many viruses are shown to be inhibited by IFNL, both in vitro and in vivo. IFNL plays a pivotal role in inhibiting viral infections at mucosal surfaces [35]. Therefore, it is imperative that pIFNLs be analyzed for their role in anti-PEDV therapeutics, which are urgently needed. The purpose of this study was to examine the relative contribution of porcine IFNLs to controlling PEDV infection in vitro.

The antiviral activities of both subtypes of pIFNLs (pIFNL1 and pIFNL3) were experimentally demonstrated. Recombinant pIFNLs were prepared by the baculovirus expression system. Here, we report that both pIFNL1 and pIFNL3 have robust antiviral activity against PEDV and VSV-EGFP infections. Virus infection experiments showed that pIFNLs could inhibit PEDV and VSV infections in different cells in a dose-dependent manner. Our results on the comparison between pIFNLs and pIFNα against PEDV infection in Vero cells also demonstrate that pIFNLs more efficiently inhibit PEDV infection in Vero cells than pIFNα, although both inhibit PEDV infection. Moreover, pIFNL3 provided better viral inhibition against PEDV infection in Vero cells than pIFNL1. Transcriptional profiling of the antiviral proteins induced by pIFNLs was detected and showed that the expressions of these ISGs genes were upregulated after the treatment of pIFNLs, especially Mx1 and IFITM3. All of these imply that IFNL preferably provides critical antiviral defenses compared with type I IFN. Collectively, we demonstrated that pIFNLs and pIFNα displayed robust antiviral activities against VSV-EGFP and PEDV infections. Moreover, our findings indicate that porcine IFNL might represent a promising therapeutic agent for PED in the future.

## 4. Materials and Methods

### 4.1. Cell Lines

Swine pulmonary alveolar macrophage (CRL2845), human embryonic kidney cells (HEK293), Madin–Darby canine kidney (MDCK), swine testis cells (ST), and African green monkey kidney cells (Vero) were cultured with Dulbecco’s Modified Eagle Medium (DMEM, HyClone, Logan, UT, USA), supplemented with 10% fetal bovine serum (FBS; Gibco, Grand Island, NY, USA), and 1% penicillin/streptomycin (Cytiva, Lewisville, TX, USA) at 37 °C with 5% CO_2_. Spodoptera frugiperda (Sf9) cells (Invitrogen, Carlsbad, CA, USA) were cultured in SF-900™ II SFM medium (Gibco, Grand Island, NY, USA) supplemented with 10% FBS and incubated at 27 °C.

### 4.2. Viruses

A strain of the Vesicular stomatitis virus that contains an enhanced green fluorescent protein gene (VSV-EGFP) was kindly provided by Prof. ZhiGao Bu from the Haerbin Veterinary Research Institute, China [36]. The porcine epidemic diarrhea virus (PEDV) strain (GenBank no. OM814174) was previously isolated in our laboratory [37].

### 4.3. Plasmid Construction and Transfection

Using specific primers, reverse transcription polymerase chain reaction (RT-PCR) was used to generate cDNA for *pIFNL1 (GenBank no. FJ455508.1)* and *pIFNL3 (GenBank no. GQ996936.1)* (Table 1). Both genes were subcloned into eukaryotic expression vector pcDNA3.1 (Invitrogen, Carlsbad, CA, USA) with the FLAG-tag at their N-terminal. Target genes were verified through DNA sequencing (Comate Bioscience Co., Ltd., Changchun, Jilin, China). HEK293 cells were transfected with plasmids. At 48 h, cells and medium supernatant were harvested. Cells were lysed using 1× radioimmunoprecipitation assay (RIPA) buffer (Merck Millipore, Temecula, CA, USA) and supplemented with phenylmethanesulfonyl fluoride (PMSF, Beyotime, Shanghai, China).

### 4.4. Analysis of Antiviral Activity of Interferon in Supernatant

Briefly, cells seeded in 12-well plates (Corning, Corning, NY, USA) were treated for 12 h with serial dilutions of supernatant preparation in 100-fold dilution in DMEM. Subsequently, cells were infected with VSV-EGFP. When obvious lesions appeared in the positive control hole, analysis of the antiviral activity by fluorescence observation, flow cytometry, and crystal violet took place.

### 4.5. Fluorescence Observation

After virus inoculation, fluorescence observation was carried out every 12 h with a Thermo Fisher Scientific EVOS M5000 microscope (Waltham, MA, USA).

### 4.6. Flow Cytometry (FCM)

At specified time points, EGFP-positive cells were collected, suspended with PBS, visualized by fluorescence microscopy, and quantified by the CytoFLEX flow cytometer (Becjkman Coulter, Brea, CA, USA).

### 4.7. Crystal Violet Staining

After washing three times with PBS, the cells were stained with 0.1% crystal violet for 15 min at room temperature. To take macrographic images—stained cells were washed with PBS and air-dried.

### 4.8. Construction of the Recombinant Baculovirus and Preparation of Protein

The pFastBac^TM^ 1 vector (Invitrogen, Carlsbad, CA, USA) was used to transfer the target gene into the baculovirus at the PH gene site. Both genes were codon-optimized according to amino acid sequences (Genscript, Nanjing, China) and synthesized. The resulting shuttle plasmids were named pFBD-pIFNL1 and pFBD-pIFNL3, respectively. The plasmids were transformed into competent DH10Bac^TM^ *E. coli* cells (Biomed, Beinjing, China), the recombinant bacmids rBD-pIFNL1 and rBD-pIFNL3 were identified by PCR (Table 1). Then, the recombinant baculovirus was rescued according to the literature [37], and the resulting recombinant baculoviruses were designated as rBV-pIFNL1 and rBV-pIFNL3, respectively. We blind-passaged the baculoviruses into Sf9 cells and kept them at −80 °C. We examined the viral titer in the third passage using the manufacturer’s instructions (Clontech, San Francisco, CA, USA). To prepare the protein, Sf9 cells in a shake flask (3 × 10^6^ cells/well) were infected with the third passage rBVs (multiplicity of infection, MOI = 1) for 72 h. The medium supernatant was collected and ultracentrifuged through a 20% sucrose cushion at 216,428× *g*, 4 °C for 2 h. The pellets were suspended in PBS and evaluated using western blot.

### 4.9. Western Blot (WB)

Cells were collected and lysed and protein concentrations were determined using a bicinchoninic acid (BCA) protein assay kit (Beyotime, Shanghai, China). Proteins were mixed with a loading buffer and denatured by boiling. Total cell extracts were prepared and separated via 10% SDA-PAGE. The proteins were transferred onto the PVDF membrane (GE Healthcare, Chicago, IL, USA) and blocked with 5% skim milk. Using specific antibodies as the primary antibodies and HRP labeled goat anti-mouse/rabbit IgG (H + L) as the secondary antibody, the protein was detected. The protein band was developed using GEGEGNOME XRQ.

### 4.10. Assay of Recombinant Interferon Titer

MDCK cells seeded in 96-well plates (Corning, Corning, NY, USA) were treated for 12 h with serial dilutions of pIFNL preparations diluted four-fold in DMEM. Subsequently, cells were infected with VSV-EGFP (the final concentration was 100 TCID_50_). When obvious lesions appeared in the positive control hole, the crystal violet staining assay was used to evaluate the interferon titer, and calculated by the Reed–Muench method.

### 4.11. Virus Infection

Cells were treated with pIFNs (100 IU/mL) for 12 h followed by infection with indicated viruses at suitable MOIs (MDCK, VSV-EGFP, 0.005 MOI; Vero, VSV-EGFP, 0.001 MOI; ST, VSV-EGFP, 0.1 MOI; Vero, PEDV, 0.1 MOI, respectively), and the DMEM served as the negative control.

### 4.12. Indirect Immunofluorescence (IFA)

Vero cells were seeded into 12-well plates at a density of 5 × 10^5^ cells/well and incubated by pIFNLs for 12 h. Then, cells were infected with PEDV at an MOI of 0.01 and cultured for a further 24 h. The cells were then fixed with ice-cold 4% paraformaldehyde (Beyotime, Shanghai, China) for 1 h and washed three times with PBS. Next, cells were blocked with 0.1% Triton X-100 (Beyotime, Shanghai, China) and 5% skim milk for 1 h. After washing a further three times with PBS, FITC-conjugated PEDV N protein-specific primary antibodies (Medgene Labs, Brookings, SD, USA) were added and incubated for 2 h at 37 °C. Cells were then washed; images were captured using a Thermo Fisher Scientific EVOS M5000 microscope.

### 4.13. TCID_50_

Vero cells were seeded into 96-well plates at 1 × 10^5^ cells per well, continuously diluting the sample 10-fold, 100 μL/well (three repetitions for each sample). After the cells had obvious CPE, the number of CPE holes under each dilution was recorded, and TCID_50_ was calculated by the Reed–Muench method.

### 4.14. qRT-PCR

Total RNA was extracted from cells (Sangon Biotech, Shanghai, China) according to the manufacturer’s instructions. cDNA was analyzed using the Fast Start Universal SYBR Green Master Mix (Roche, San Francisco, CA, USA) for quantitative PCR (qPCR). The primer sequences are listed in Table 1. Relative quantities were calculated and normalized to β-actin using the 2^−ΔΔCT^ method.

### 4.15. Statistical Analyses

With the one-way analysis of variance (ANOVA; two-tailed, confidence intervals (CI) 95%), the statistical analysis was conducted using GraphPad 8.0 (GraphPad Software, San Diego, CA, USA), as indicated by the *p*-value. The results were statistically significant at *p* < 0.05. For each separate set of assays, at least three independent experiments were evaluated. The results are expressed as the mean ± standard deviation (SD).

## 5. Conclusions

In conclusion, the baculovirus system was used to construct and express pIFNLs; pIFNLs not only could inhibit PEDV but also VSV infection in different cells in a dose-dependent manner. The expressions of ISGs genes were upregulated after the treatment of pIFNLs, especially Mx1 and IFITM3.

## Figures and Tables

**Table 1:** Primers in this study.

Class	Primer	Sequence (5′-3′)	Length (bp)	Gene Name
PCR	pIFNL1F	GCTAGCGCCACCATGGAT- TACAAGGATGACGACGATAAGATGG- TATGCTACGGGGTCAC	576	*pIFNL1*
	pIFNL1R	AAGCTTCTAAGTGCAATCCTCGCGC		
	pIFNL3F	GCTAGCGCCACCATGGAT- TACAAGGATGACGACGATAAGATGG- TATGCTACGGGGTCAC	588	*pIFNL3*
	pIFNL3R	AAGCTTTCACTGTGCTGGGGACTG		
qRT- PCR	PKRR	TTGCGAGAAGGTAGAGCGTG	90	*pPKR*
	PKRF	TCATTCCCATCCCAGCAACC		
	Mx1F	CAGAGGCAGCGGAATTGTGA	99	*pMx1*
	Mx1R	TCCCGGTAACTGACTTTGCC		
	IFITM3F	CATGGAGGACCCCCAACATA	336	*pIFITM3*
	IFITM3R	GCAAACGATGATGAACGCAA		
	ISG15F	TCCTGTTGATGGTGCAAAGC	235	*pISG15*
	ISG15R	ATACACGGTGCACATAGGCT		
	β-actin F	CTTCCTGGGTAGGTGTCGG	160	*pβ-actin*
	β-actin R	CGTCGCACTTCATGATCGAG		

## Data Availability

All data generated or analyzed during this study are included in this published article.
